# Dynamics of SARS-CoV-2 Mutations in Wastewater Provide Insights into the Circulation of Virus Variants in the Population

**DOI:** 10.3390/ijms26094324

**Published:** 2025-05-01

**Authors:** Sara Mesquita Costa, Maria Clara da Costa Simas, Luciana Jesus da Costa, Rosane Silva

**Affiliations:** 1 Instituto de Biofísica Carlos Chagas Filho, Universidade Federal do Rio de Janeiro, Rio de Janeiro 21941-902, RJ, Brazil; costasm@biof.ufrj.br (S.M.C.); clara.simas@biof.ufrj.br (M.C.d.C.S.); 2 Instituto de Microbiologia Paulo de Góes, Universidade Federal do Rio de Janeiro, Rio de Janeiro 21941-902, RJ, Brazil; ljcosta@micro.ufrj.br

**Keywords:** wastewater-based epidemiology, coronavirus, nested-PCR, next-generation sequencing

## Abstract

SARS-CoV-2 high transmission and genomic mutations result in the emergence of new variants that impact COVID-19 vaccine efficacy and virus transmission by evading the host immune system. Wastewater-based epidemiology is an effective approach to monitor SARS-CoV-2 variants circulation in the population but is a challenge due to the presence of reaction inhibitors and the low concentrations of SARS-CoV-2 in this environment. Here, we aim to improve SARS-CoV-2 variant detection in wastewater by employing nested PCR followed by next-generation sequencing (NGS) of small amplicons of the S gene. Eight SARS-CoV-2 wastewater samples from Alegria Wastewater Treatment Plant, in Rio de Janeiro, Brazil, were collected monthly from February to September 2021. Samples were submitted to virus concentration, RNA extraction and nested PCR followed by NGS. The small amplicons were used to prepare libraries for sequencing without the need to perform any fragmentation step. We identified and calculated the frequencies of 29 mutations matching the Alpha, Beta, Gamma, Delta, Omicron, and P.2 variants. Omicron matching-mutations were detected before the lineage was classified as a variant of concern. SARS-CoV-2 wastewater sequences clustered with SARS-CoV-2 variants detected in clinical samples that circulated in 2021 in Rio de Janeiro. We show that sequencing of selected small amplicons of SARS-CoV-2 S gene allows the identification of SARS-CoV-2 variants matching mutations and their frequencies’ calculation. This approach may be expanded using customizing primers for additional genomic regions, in order to differentiate current variants. Approaches that allow us to learn how variants emerge and how they relate to clinical outcomes are crucial for our understanding of the dynamics of virus variants circulation, providing valuable data for public health management.

## 1. Introduction

As of March 2025, the COVID-19 resulted in more than 777 million reported cases and more than 7 million confirmed deaths globally [1]. SARS-CoV-2 high transmission and mutation occurrence in the virus genome result in the emergence of new variants that impact COVID-19 vaccine efficacy and virus transmission by evading the host immune system [2]. The World Health Organization (WHO) have classified the variants in three groups: variants of concern (VOCs), variants of interest (VOIs), and variants under monitoring (VUMs) [3].

VUMs are variants with genetic changes that are suspected to affect virus characteristics and spread more than other circulating variants, but evidence of their phenotypic or epidemiological impact remains unclear [4]. VOIs have genetic changes that are predicted to affect virus transmissibility, virulence, antibody evasion, susceptibility to therapeutics, and detection. VOIs may also show high prevalence in the population, having an epidemiological impact suggestive of an emerging risk to global public health [4]. P.2 was a VOI first detected in the clinical setting in April 2020 and was widespread in Rio de Janeiro, Brazil [3,5]. VOCs are variants associated with a more severe clinical presentation, or changes in COVID-19 epidemiology that cause a substantial impact on the ability of health systems to provide care to patients with COVID-19 or others illness. They represent a burden on the public health system. Additionally, VOCs are variants known to significantly decrease the effectiveness of available vaccines in protecting against severe disease [4]. The following variants were assigned as VOCs and were first detected in clinical samples: Alpha (B.1.1.7) in the United Kingdom (September 2020), Beta (B.1.351) in South Africa (October 2020), Gamma (P.1) in Brazil (January 2021), Delta (B.1.617.2) in India (December 2020), and Omicron (B.1.1.529) in South Africa (November 2021) [6,7,8,9,10]. The Omicron variant has already many sublineages that spread globally [11].

SARS-CoV-2 variants have many polymorphisms, mostly in the S protein coding gene. The Spike protein comprises an S1 subunit facing the surface of the membrane and an S2 transmembrane subunit. The S1 subunit is responsible for binding to the receptor on the target cell, whereas the S2 is involved in the fusion of the viral and host membrane cell, playing a crucial role in the entry of the virus into target cells [12,13]. The S1 subunit encompasses the N-terminal domain (NTD) and the receptor binding domain (RBD), both of which harbor numerous polymorphisms [14]. Since the S gene harbors many mutations and some of them differ among SARS-CoV-2 variants, nucleotide sequencing of the S gene is used for identifying virus variants [15,16].

Considering that SARS-CoV-2 RNA has been detected in feces of infected people, wastewater-based epidemiology (WBE) serves as a valuable tool for assessing the prevalence of infections within the population [17,18]. Indeed, WBE is effective in monitoring SARS-CoV-2 in the population. This approach has been employed to quantify SARS-CoV-2 and identify variants in sewage [19,20]. However, genome detection by WBE is challenging due to the presence of reaction inhibitors and the low concentrations of SARS-CoV-2 in sewage [21,22]. Therefore, the present study aims to improve SARS-CoV-2 variant detection in wastewater by employing nested-PCR followed by next-generation sequencing of selected small amplicons of the S gene. Samples were collected between February and September 2021 in a major Wastewater Treatment Plant of Rio de Janeiro, Brazil. The results provide valuable insights into the prevalence and spread of the virus in the region. Our analysis identified and estimated the frequency of SARS-CoV-2 variants mutations in wastewater samples, which were then compared to the occurrence of clinical cases at the time. Our approach successfully identified SARS-CoV-2 variants, overcoming challenges posed by reaction inhibitors and low virus concentration.

## 2. Results

### 2.1. SARS-CoV-2 S Gene Fragments Are Efficiently Amplified in Wastewater Samples

**Figure 1:**
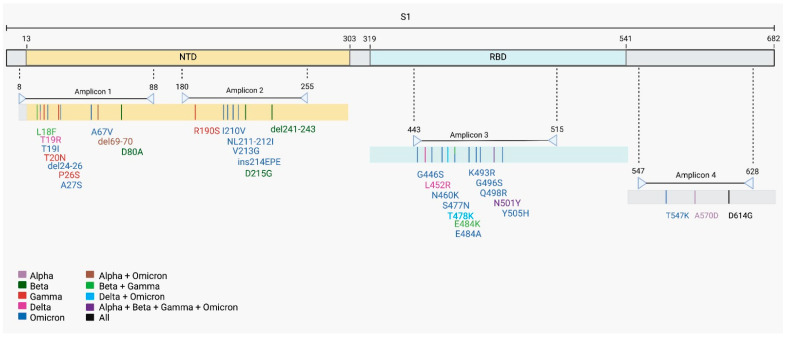
SARS-CoV-2 Spike mutations and indels associated with variants of concern. SARS-CoV-2 Spike mutation and indels covered by the four selected regions of nested-PCR. Number above the S1 subunit indicates the amino acid position of the S1 domains. The domain division was obtained in [23]. Numbers and arrows above colored bars indicate amino acid position and the amplification region in the spike protein. Spike mutations were obtained in [24]. See materials and methods section for the primer pairs used to amplify each amplicon. Omicron mutations refer to BA.1 and BA.2 sublineages. Created with BioRender.com (accessed on 21 April 2024).

**Figure 2:**
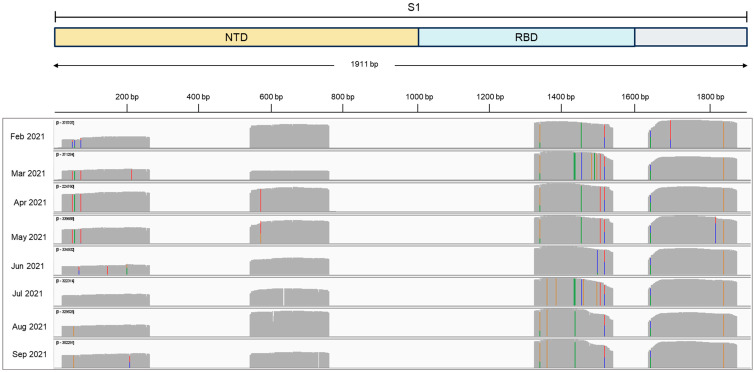
Sequencing of SARS-CoV-2 S amplicons. SARS-CoV-2 S reads aligned to SARS-CoV-2 reference sequence (Wuhan S gene) were loaded in IGV v. 2.16.2. Image shows the expected four Spike fragments. Gray bars indicate nucleotides similarity to the reference sequence. Colorful bars indicate mutation sites. Bars level indicates read counts. S1: SARS-CoV-2 S1 subunit of S gene; NTD: N-terminal domain, RBD: receptor-binding domain.

Alegria Wastewater Treatment Plant (WWTP) samples, collected between February and September of 2021, showed a low amount of SARS-CoV-2 genomic RNA, as revealed by the Cts obtained for each sample by the SARS-CoV-2 diagnostics RT-qPCR assay (Table 1). To increase the amount of SARS-CoV-2 template used for sequencing, we performed a nested-PCR of selected regions in the S gene that generated amplicons of around 230 bp. These regions harbor Spike mutations and indels that were associated with a variant of concern (Figure 1). All four S regions from the eight wastewater samples were successfully amplified by nested-PCR (Figure S1) and sequenced using the Ion Torrent Platform (Figure 2). Results show mutations at different sites over eight months, each month corresponding to a unique sample collection, that matched virus variants that were considered variants of concern (VOCs) at the time of sample collection (Figure 2). Our results show that nested-PCR efficiently amplified SARS-CoV-2 S regions that harbor mutation sites, as confirmed by next-generation sequencing.

### 2.2. Identification of 29 Mutations in SARS-CoV-2 S Gene

To investigate the mutations associated with SARS-CoV-2 S variants, we searched for Single Nucleotide Polymorphisms (SNPs) and found 29 different SARS-CoV-2 S mutations in Alegria WWTP samples (Table 2). Mutations matched those found in SARS-CoV-2 Alpha, Beta, Gamma, Delta, Omicron, and P.2 variants. We also identified mutations that were not yet described at the time of sample collection. Mutations E484K and D614G predominated in February, less frequent mutations that match Gamma and Omicron variants were also observed during this period. Mutations found in samples from March matched all variants of concern; Gamma and Omicron mutations predominated during this period. In samples from April and May, we detected five mutations that matched Gamma while three matched Omicron variants. Curiously, Gamma matching mutations were fewer in February, but were abundant in March, April, and May. In June, we detected mutations that were not described at the time of sample collection. In July, August, and September, we detected mutations that matched Delta and Omicron variants (Table 2). Omicron matching mutations were detected in all months at varied frequencies. Interestingly, we first detected an Omicron matching mutation in February 2021, although the Omicron variant was first documented in a clinical sample months later, in November 2021 [3]. Because in June we detected four mutations that had not been described at the time that this article was under preparation, while in March and July many Omicron matching mutations were detected, we performed Sanger sequencing to check their sequences. Sanger results confirmed the NGS results. Together, these results indicate that it is possible to monitor SARS-CoV-2 variant changes through wastewater using next-generation sequencing of selected small fragments of the S gene.

### 2.3. SARS-CoV-2 Variants Classification in Wastewater

**Figure 3:**
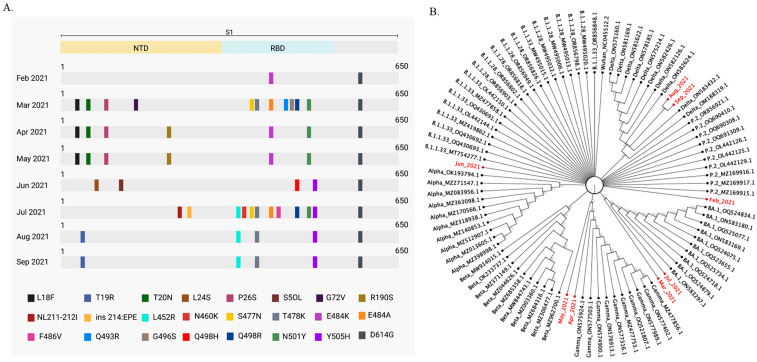
SARS-CoV-2 variants classification in wastewater. (**A**). SARS-CoV-2 single nucleotide polymorphism (SNP) and indels detected in Alegria WWTP. Graphic shows variant frequencies above 55%. (**B**). Phylogenetic tree was generated from nucleotide alignments based on SNP and indels shown in (**A**). The tree was built in CLC Genomic Workbench v.24.0.1 with Neighbor Joining construction method, HKY nucleotide substitution model and 1000 replicates of bootstrap. Only bootstrap above 55% is shown. Wastewater samples are highlighted in red. NTD: N-terminal domain, RBD: receptor-binding domain.

To identify SARS-CoV-2 predominant variants circulating in wastewater from February to September 2021 in Rio de Janeiro, we aligned our sequenced samples to SARS-CoV-2 variant sequences from 2021, available at the Virus Variation Resource at the National Center for Biotechnology Information [25]. A phylogenetic tree was built based on this alignment that contemplated only mutations with frequencies above 55%. March and July were the months when we found the greater number of mutations. Omicron mutations were in high frequency during these months. Mutations previously associated with VOCs were identified in all months (Figure 3A). Sequenced samples were compared to B.1.1.33, B.1.1.28, Alpha, Beta, Delta, Gamma, Omicron (BA.1), and P.2 sequences. We analyzed all VOCs circulating in Brazil in 2021 and the P.2 variant. P.2 was a SARS-CoV-2 variant of interest (VOI) that emerged in April 2020 and had high circulation in Rio de Janeiro [3,5]. The Wuhan SARS-CoV-2 reference sequence and the early lineages B.1.1.33 and B.1.1.28 were also included in the analysis. SARS-CoV-2 sequence clusters are shown in Figure 3B. As expected, April and May wastewater samples clustered with Gamma sequences, while August and September samples clustered with Delta sequences. On the other hand, March and July wastewater samples clustered with Omicron (BA.1) sequences, although Omicron became a VOC only in November 2021. Together, these results show that the dynamics of SARS-CoV-2 variants circulation may be inferred by phylogenetic analysis that included the wastewater predominant mutations found in clinical sequences available on the database [26].

### 2.4. SARS-CoV-2 Mutations Found in Wastewater Samples Match SARS-CoV-2 Variants in Clinical Samples

**Figure 4:**
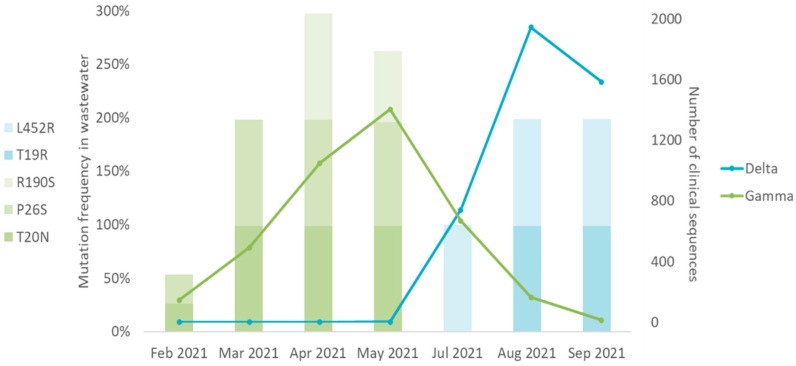
SARS-CoV-2 mutations found in wastewater samples match SARS-CoV-2 variants in clinical samples. Green and blue bars indicate SARS-CoV-2 Gamma and Delta mutations identified in wastewater, respectively. Wastewater results are shown as percentages (left axis). Green and blue lines show SARS-CoV-2 Gamma and Delta variants detected in clinical samples, respectively. On the right axis are the absolute number of clinical samples sequences. Clinical sample data were accessed in [26]. Data were obtained from the Rio de Janeiro dashboard. Omicron matching mutations were omitted in the analysis.

To investigate if the SARS-CoV-2 variant in wastewater reflected the findings obtained from clinical samples in the same period, we compared the frequency of the variant-matching mutation detected in wastewater with clinical SARS-CoV-2 variant sequences. Only mutations that were unique to a single variant were considered. SARS-CoV-2 clinical sequences were obtained in [26] enabled by data from GISAID. Omicron matching mutations detected in wastewater samples were omitted in the analysis, since there were no clinical sequences available during the corresponding period—February to September 2021. Data analyses showed that Gamma and Delta variants were the prevalent variants circulating in Rio de Janeiro between February and September 2021 (Figure 4). Gamma matching mutations were prevalent between February and May 2021, the period when Gamma was the major variant identified in clinical samples. Delta matching mutations were prevalent between July and September 2021, the period when Delta was prevalent in clinical samples (Figure 4). These results suggest that wastewater samples reflect the distribution profile of SARS-CoV-2 variants found in clinical samples through time.

## 3. Discussion

We were able to identify mutation-matching SARS-CoV-2 variants and estimate their frequencies in wastewater samples. Alegria Wastewater Treatment Plant (WWTP) samples from Rio de Janeiro, Brazil yielded a low amount of SARS-CoV-2 genomic RNA (Table 1), which could compromise the whole genome sequencing. In these situations, previous amplification of target regions, such as of a gene or part of a gene, can improve the sequencing performance [27,28]. The number and genome location of target regions need to be carefully selected to detect sufficient mutations to identify variants. Thus, we carried out nested-PCR based on four SARS-CoV-2 S gene regions (Figure 1). The small size of the amplicons (around 230 bp) lets us prepare the libraries for sequencing without the need to perform any fragmentation step. The removal of the fragmentation step reduces sequencing artifacts and enzymatic bias, such as insertion and deletion of nucleotides resulting from physical and enzymatic shearing. This strategy improves library construction and the quality of the next-generation sequencing (NGS) [29,30].

Nested-PCR efficiently amplified the four SARS-CoV-2 S regions from Alegria WWTP samples (Figure S1). Additionally, amplicons harboring virus mutation sites were efficiently sequenced in the Ion Torrent Platform (Figure 2), showing that sequencing of small fragments of the S gene previously amplified is a successful approach to adopt when working with wastewater samples that have low amounts of SARS-CoV-2 genomic RNA.

Between February and September 2021, we found 29 SARS-CoV-2 S mutations in Alegria WWTP samples that matched P.2 and were considered VOCs at the time (Table 2). Studies show that the frequency of the Alpha mutation varied in wastewaters in England and Japan, between October 2020 and January 2021 [31]. Similarly, we detected Gamma mutations at various frequencies between February and May 2021 in Rio de Janeiro wastewater samples. Gamma mutations were detected at low frequency in February 2021, and then at increasing frequencies in the following months, with the predominant variant found in April and May 2021 (Table 2, Figure 3). Our finding indicates that by adopting next-generation sequencing of selected small fragments of the S gene, it is possible to identify SARS-CoV-2 variant mutations that appear at low frequencies in wastewater before they become predominant in the population.

Studies show that VOCs and mutations associated with VOCs can be identified in wastewater in periods between 2 months to 2 weeks before it is detected in clinical samples [32,33,34]. We identified mutations matching those found in the Omicron variant (BA.1 and BA.2 sublineages) nine months before they were described in the first clinical sample, suggesting the potential of wastewater surveillance to anticipate variant detection (Table 2) [3]. Omicron matching mutations were detected in high frequency in wastewater samples in March and July 2021. At the same time, Gamma and Delta matching mutations were also detected in high frequency, and the latter were the ones that became predominant in April and August 2021, respectively, while Omicron was later established in December 2021 (Table 2; Figure 3) [3]. Shempela and co-worker demonstrated that B.1.1.529, the first Omicron lineage described, may not have been completely replaced by newer Omicron subvariants and thus could be circulating and possibly contributing to the community transmission of SARS-CoV-2 [35]. As such, SARS-CoV-2 variants and subvariants co-circulation seem to be common.

Studies show that Omicron replication and infectivity in Caco-2 cells, an epithelial cell derived from colon tissue, is lower than that observed in Gamma and Delta variants [36]. Gamma and Delta high kinetic replication in colon tissue may explain the prevalence of these variants in wastewater, which is an environment known to be predominated by feces. It is possible that Omicron emerged at the same time as Gamma and Delta variants, but the latter variants initially predominated. The vaccination program that started in March 2021 in Brazil could have contributed to further establish Omicrons as a major variant circulating in Brazil, since vaccination was more effective against Gamma and Delta [37,38,39].

Phylogenetic analysis of SARS-CoV-2 mutations detected in wastewater samples and SARS-CoV-2 sequences from Virus Variation Resource hosted by the National Center for Biotechnology Information allowed us to infer about variant circulation in wastewater between February and September 2021 (Figure 3B). Wastewater samples from February clustered with P.2 sequences (Figure 3B). Indeed, the P.2 variant was prevalent during this period in Rio de Janeiro [40]. Wastewater samples collected between April and May 2021 clustered with Gamma sequences, and wastewater samples from August to September clustered with Delta sequences, which agreed with the variants more prevalent in clinical samples during these periods (Figure 4), indicating that wastewater samples reflect the distribution of SARS-CoV-2 variants in the population.

While this article was under preparation, new Omicron subvariants emerged that could not be investigated by our approach, because it would require the amplification and sequencing of additional genomic regions [41]. In fact, we were not able to predict which one would be the next variant to be established in the population because the spread of a new variant depends on many factors that cannot be inferred based solely on the frequency of a given mutation at a given time. Nevertheless, we did detect Omicron (BA.1 and BA.2) mutations in March of 2021, and the variant was established later, in December of the same year (Figure 3A) [3]. The limited number of sewage samples is acknowledged as a constraint, and efforts to enhance this aspect would be beneficial. Despite this, the intrinsic properties of sewage samples—such as low RNA virus concentrations and the presence of PCR inhibitors—often result in studies that assess fewer samples when using NGS [31]. Our study identifies and calculates mutation frequencies for Alpha, Beta, Gamma, Delta, Omicron, and P.2 variants in sewage samples. Furthermore, it illustrates the dissemination of these variants within the local human population.

The approach used in this study may be applied to detect SARS-CoV-2 mutation-matching variants in wastewater samples with low amounts of genomic RNA. The approach may be expanded by designing customized primers for additional genomic regions to identify new variants, contributing to SARS-CoV-2 wastewater monitoring. New sublineages of SARS-CoV-2 are continuously emerging, and another pandemic caused by other pathogens is a real threat. Therefore, approaches that allow us to learn how variants emerge and how they relate to clinical outcomes are crucial for our understanding of the pathogen circulation, providing valuable data for public health management.

## 4. Materials and Methods

### 4.1. Samples Information

Eight SARS-CoV-2 positive wastewater samples were obtained from Estudo Monitora Corona coordinated by Professor Isaac Volshan Jr. from the Department of Water Resources and Environment from Polytechnic Institute of Federal University of Rio de Janeiro, Brazil. Single samples were manually collected monthly on a timely basis between February 2021 and September 2021 from Alegria Wastewater Treatment Plant (WWTP) located in the metropolitan area of Rio de Janeiro, Brazil. Alegria was the biggest WWTP operated by Companhia Estadual de Águas e Esgotos (CEDAE), the major city’s sanitation company at the time of this study. Alegria WWTP receives sewage from 1,165,042 people living in 45 neighborhoods. Wastewater samples were kept at 4 °C and were processed for up to 72 h.

### 4.2. Virus Concentration

Wastewater samples were pasteurized at 60 °C for 90 min to ensure safety for the laboratory personnel [42,43]. After inactivation, 40 mL of samples were filtered in 0.22 μm polyethersulfone membrane (ref. K18-230, Kasvi, Curitiba, PR, Brazil) to remove bacterial cells and debris. Filtered samples were precipitated with polyethylene glycol 8000 (10% [wt/vol]; Promega, Madison, WI, USA) and NaCl (0.4 M; Isofar, Rio de Janeiro, RJ, Brazil). The mixtures were shaken at room temperature until polyethylene glycol was fully dissolved and centrifuged at 9000× *g* for 2 h. The viral pellet was resuspended in 1.0 mL of PBS and proceeded to RNA extraction.

### 4.3. RNA Extraction and SARS-CoV-2 RT-qPCR

Viral RNA was extracted from 0.2 mL of viral concentrate with Bio Gene Viral DNA/RNA Extraction (ref. K204-4, Bioclin, Belo Horizonte, MG, Brazil) following manufacturer’s protocol. RNA was eluted in 50 μL of DNAse/RNAse free water and quantified by RT-qPCR using primers and probes targeting the SARS-CoV-2 N gene. Primers and probes were obtained from a previous study “Estudo Monitora Corona” shown in Table S1. Reactions were performed with 5 μL of extracted RNA (total RNA range of 14 to 25 ng) in a final volume of 25 μL using AgPath-ID™ One-Step RT-PCR Reagents (Thermo Fisher Scientific, Waltham, MA, USA) following manufacturer’s instructions. Quality controls included a positive control (SARS-CoV-2 clinical positive sample) and a negative control (DNAse/RNAse free water). The RT-qPCR was carried out for 40 cycles on ViiA 7 Real-Time PCR System (Thermo Fisher Scientific, Waltham, MA, USA) based on the following program: 45 °C for 10 min; 95 °C for 10 min; and 40 cycles of 95 °C for 15 s and 60 °C for 45 s.

### 4.4. SARS-CoV-2 S Gene Targeted Amplification

**Figure 5:**
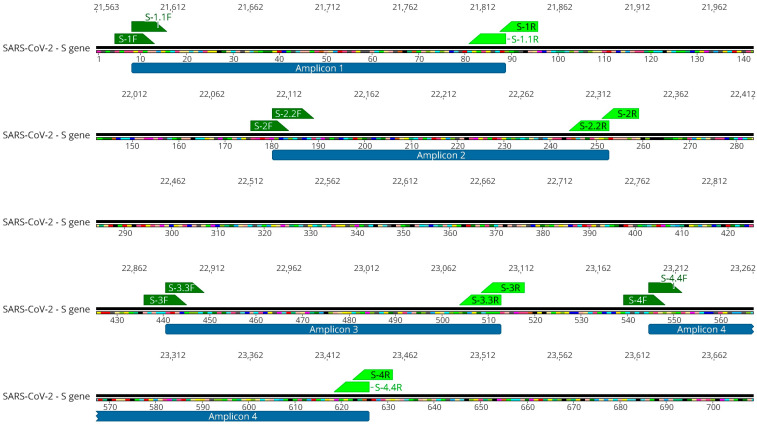
Primers for nested-PCR to SARS-CoV-2 S gene and amplicons regions. Numbers below the sequence refer to amino acid position in the S gene and numbers above the sequence refer to nucleotide position in the SARS-CoV-2 genome. Green annotations indicate primers binding sites. Blue annotations refer to amplicon regions.

Nested-PCR was performed to amplify four selected Spike regions. The selected regions for amplification cover mutation and indels present in major variants of concern that circulated in 2021. Primers for SARS-CoV-2 S gene were designed using the S gene from Wuhan reference genome (GenBank: NC_045512.2) using Geneious Prime Software v. 2022.2 (Figure 5). Primers sequences are shown in Table 3.

First-strand cDNA was synthesized using SuperScript III Reverse Transcriptase (Thermo Fisher Scientific, Waltham, MA, USA) with random primer and using 5.0 μL of RNA (total RNA range of 14 to 25 ng). First PCR reaction was performed using 4.0 μL of cDNA in a final volume of 25 μL, using 2 mM MgCl_2_, 0.2 mM dNTP, 0.2 μM of primers, 5% DMSO, and 1.25 U DNA Polymerase (Go Taq Flexi DNA Polymerase, Promega, Madison, WI, USA). The PCR conditions were as follows: 95 °C for 2 min; 15 cycles of 95 °C for 30 s, 50 °C for 30 s and 72 °C for 30 s; 20 cycles of 95 °C for 30 s, 53 °C for 30 s and 72 °C for 30 s; and final extension 72 °C for 5 min. Nested-PCR was performed using 2 μL of first PCR product in a final volume of 25 μL, using 2 mM MgCl_2_, 0.2 mM dNTP, 0.2 μM of primers, and 1.25 U DNA Polymerase (Go Taq Flexi DNA Polymerase, Promega, Madison, WI, USA). The nested-PCR conditions were as follows: 95 °C for 2 min; 35 cycles of 95 °C for 30 s, 53 °C for 30 s and 72 °C for 30 s; and final extension 72 °C for 5 min. DNAse/RNAse free water was used as the negative control. The amplified PCR product quality was visualized in 4150 TapeStation System (Agilent Technology, Santa Clara, CA, USA) using the Agilent High Sensitivity D1000 ScreenTape Kit following manufacturer’s instructions.

### 4.5. Next-Generation Sequencing (NGS)

Nested-PCR amplicons were purified using Agencourt AMPure XP Reagent (Beckman Coulter, CA, USA) and quantified by Qubit^TM^ dsDNA BR Assay (Invitrogen, Waltham, MA, USA). A total of 25 ng of the purified amplicons corresponding to each of the four S gene regions of the same sample were combined to a final concentration of 100 ng. NGS was achieved using the Ion Plus Fragment Library Kit (Thermo Fisher Scientific, Waltham, MA, USA), following manufacturing instructions (MAN0006846). Quantification of the libraries was obtained using the Ion Library TaqMan™ Quantitation Kit (Thermo Fisher Scientific, Waltham, MA, USA). The libraries were pooled at a concentration of 40 pM and run on the Ion Chef™ system (Thermo Fisher Scientific, Waltham, MA, USA) using Ion 510, 520 & 530 Kits (Thermo Fisher Scientific, Waltham, MA, USA) and run into a 530 chip in an Ion S5™ System genetic sequencer (Thermo Fisher Scientific, Waltham, MA, USA).

### 4.6. Data Analysis

The sequencing reads generated were mapped to a SARS-CoV-2 reference genome Wuhan (GenBank: MN908947) using the Ion Browser software included in the Torrent Suite 5.18.1. and visualized in Integrative Genomics Viewer (IGV Version 2.16.2) (Broad Institute, Cambridge, MA, USA) [44]. Reads were trimmed using BBDuk Adapter/Quality Trimming Version 38.84 (by Brian Bushnell) available in Geneious Prime Software v.2023.2. The trim parameter was minimum quality = 20; minimum length = 200 bp. Trimmed reads were mapped to a SARS-CoV-2 S gene reference genome (GenBank: NC_045512.2) and Single Nucleotide Polymorphisms (SNP_S_) achieved using Geneious Prime Workflow “Map reads then find variations/SNPs”. The parameter setting was: Bowtie2 mapper v. 7.2.2 with a highest sensitivity local alignment type; minimum variant frequency = 0.1, find variants only inside CDS, standard analyze effect of variants on translations, assumed quality of bases without quality = 20 [45]. Consensus sequences were aligned using MAFFT alignment v7.490 with Geneious Prime default [46,47]. The four amplicons sequenced were manually concatenated for each sample.

### 4.7. Phylogenetic Tree

A total of 80 SARS-CoV-2 nucleotide sequences from B.1.1.33, B.1.1.28, P.2, Alpha, Beta, Gamma, Delta, and Omicron BA.1 variants and Wuhan SARS-CoV-2 reference sequence were downloaded from Virus Variation Resource hosted by the National Center for Biotechnology Information (NCBI) [25]. We used filters: Geographic Region: Brazil; Nucleotide Completeness: complete. Selected sequences are shown in Table 4.

Sequences were aligned using MAFFT alignment, followed by S gene extraction and realignment. Sequences were cut and concatenated to harbor only the four regions of Spike that correspond to our approach of small S amplicons and used to build a phylogenetic tree. Mutations above 55% frequency were contemplated for tree analyses. A model test was run in CLC Genomic Workbench v.24.0.1, and the HKY model was recommended. A phylogenetic tree was built in CLC Genomic Workbench v.24.0.1 with parameters as follows: Neighbor Joining construction method, HKY nucleotide substitution model, number of substitution rate categories equal 4, gamma distribution parameter equal 1, and 1000 replicates of bootstrap.

## 5. Conclusions

Nested-PCR followed by next-generation sequencing of selected small amplicons of the S gene improves SARS-CoV-2 variant detection in wastewater and allows monitoring of SARS-CoV-2 variant changes through time. Our approach allowed us to detect Omicron matched mutations nine months before they were described in the first clinical sample, suggesting the potential of wastewater surveillance to anticipate variant’s detection. Furthermore, wastewater samples reflect the distribution profile of SARS-CoV-2 variants found in clinical samples. Therefore, SARS-CoV-2 genome monitoring in wastewater contributes to our understanding of the virus dynamic circulation in the population, providing helpful data for the public health system.

## Figures and Tables

**Table 1:** SARS-CoV-2 RNA quantification by RT-qPCR.

Sample	Ct
February 2021	32.3
March 2021	33.4
April 2021	32.4
May 2021	31.8
June 2021	28.4
July 2021	34.1
August 2021	31.3
September 2021	31.2

Ct = Cycle Threshold.

**Table 2:** Dynamics of SARS-CoV-2 mutation frequencies from February to September 2021 in Wastewater Treatment Plant in Rio de Janeiro, Brazil.

	NTD												RBD																
	L18F	T19R	T20N	L24S	P26S	S50L	I68V	G72V	T76A	R190S	NL211-212I	ins214EPE	G446S	L452R	N460K	S477N	T478K	E484A	E484K	F486V	Q493R	S494A	G496S	Q498H	Q498R	N501Y	Y505H	T547K	D614G
February 2021	26		26		27								26						100								48	38	100
March 2021	99		99		99			99					31			100	99	100			99		100		100	100	53	41	100
April 2021	99		99		99					99			28						98							100	49	31	100
May 2021	99		99		97					66			24						100							100	51	52	100
June 2021				57		98	31															17		96			55	34	100
July 2021											98	97	17	100	100	100	100	100		99					100	100	71	47	100
August 2021		99							11				26	100			99										58	35	100
September 2021		99											28	100			99										56	42	100

NTD: N-terminal domain; RBD: receptor binding domain. Number in the box indicates the percentage of amino acid change found in wastewater samples compared to the Wuhan reference sequence. Omicron refers to BA.1 or BA.2 mutations, except for S50L mutation that refer to BA.2.86 and JN.1, and F486V, that refer to BA.4. ■ Alpha/Beta/Gamma/Omicron; ■ Delta/Omicron; ■ Beta/Gamma; ■ Omicron; ■ Beta/Gamma/P.2; ■ VOCs or VOIs; ■ Delta; ■ Not previously described; ■ Gamma.

**Table 3:** Primers for nested-PCR to SARS-CoV-2 S gene.

Primer Name	Nucleotide Sequence (5′-3′)	Orientation	Reaction	Amplicon Size (bp)
S-1F	CTTGTTTTATTGCCACTAGTCTCTAG	Sense	First PCR	273
S-1R	CARTGGAAGCAAAATAAACACCATC	Anti-sense		
S-1.1F	GCCACTAGTCTCTAGTCAGTGTG	Sense	Nested-PCR	241
S-1.1R	CATCATTAAATGGTAGGACAGGGT	Anti-sense		
S-2F	CTTATGGACCTTGAAGGAAAACAGG	Sense	First PCR	259
S-2R	GTCCAACCTGAAGAAGAATCACCA	Anti-sense		
S-2.2F	AGGAAAACAGGGTAATTTCAAAAATCT	Sense	Nested-PCR	226
S-2.2R	CACCAGGAGTCAAATAACTTCTATGT	Anti-sense		
S-3F	GGAATTCTAACAAKCTTGATTCTAAGGT	Sense	First PCR	245
S-3R	GAAGTTCAAAAGAAAGTACTACTACTCT	Anti-sense		
S-3.3F	CTTGATTCTAAGGTTRGTGGTAATT	Sense	Nested-PCR	216
S-3.3R	GTACTACTACTCTGTATGGTTGGTRAC	Anti-sense		
S-4F	CAATTTCAACTTCAATGGTTTAAMAGG	Sense	First PCR	275
S-4R	GGAGTAAGTTGATCTGCATGAATAGC	Anti-sense		
S-4.4F	GGTTTAAMAGGCACAGGTGTTC	Sense	Nested-PCR	244
S-4.4R	GCATGAATAGCAACAGGGACTTC	Anti-sense		

**Table 4:** Sequences downloaded from NCBI for phylogenetic tree construction.

SARS-CoV-2 Lineage	Sequence ID
Wuhan	NC_045512.2
B.1.1.33	OL442150.1; MZ477858.1; OQ430691.1; OL442144.1; MZ419862.1; OQ430692.1; OQ430693.1; MT754277.1; OR856848.1
B.1.1.28	MW495020.1; OR856798.1; MW495013.1; MW495006.1; MW495012.1; MW495015.1; OR856826.1; OR856949.1; OR856818.1; OR856802.1; OR856903.1
P.2	MZ169915.1; MZ169917.1; MZ169916.1; OL442129.1; OL442125.1; OL442126.1; OQ691309.1; OQ690308.1; OQ690410.1; OR856921.1
Alpha	OK193794.1; MZ271547.1; MZ083956.1; MZ363098.1; MZ170566.1; MZ318938.1; MZ140853.1; MZ512907.1; MZ013605.1; MZ398998.1
Beta	MZ685358.1; MW844743.1; MZ003360.1; MZ684316.1; MZ306477.1; MW914015.1; OK233737.1; MZ371149.1; MZ044626.1; MZ962700.1
Gamma	ON575924.1; ON577503.1; ON574900.1; ON576913.1; ON577316.1; MZ477753.1; OQ521807.1; ON577989.1; ON577402.1; MZ477856.1
Delta	ON582624.1; ON583432.1; OM188319.1; ON582126.1; ON582426.1; ON575214.1; ON578185.1; ON581622.1; ON581169.1; ON575160.1
Omicron (BA.1)	ON583297.1; OQ524879.1; OQ524718.1; OQ525734.1; OQ523655.1; OQ524075.1; ON583169.1; OQ525077.1; ON583180.1; OQ524834.1

## Data Availability

DNA sequences generated during this study are available in the NCBI database (https://www.ncbi.nlm.nih.gov/nuccore/. accessed on 4 October 2024) under the following accession IDs: PP579733, PP579734, PP579735, PP579736, PP579737, PP579738, PP579739, PP579740.

## Supplementary Materials

The following supporting information can be downloaded at: https://www.mdpi.com/article/10.3390/ijms26094324/s1.
