# Supplementary material for: Dynamics of SARS-CoV-2 Mutations in Wastewater Provide Insights into the Circulation of Virus Variants in the Population
Source: Int J Mol Sci. 2025 May 1;26(9):4324. doi: 10.3390/ijms26094324 (PMC12072199; doi:10.3390/ijms26094324)
Supplement: Supplementary file 1 [file ijms-26-04324-s001.zip › ijms-3557624-supplementary.pdf]

Supplementary materials

Table S1: SARS-CoV-2 primers and probes for RT-qPCR.

Primer/Probe	Concentration	Sequence	Reference
**2019-nCoV_N1-F**	400nM	GAC CCC AAA ATC AGC GAA AT	
**2019-nCoV_N1-R**	400nM	TCT GGT TAC TGC CAG TTG AAT CTG	Lu et al.,2020
**2019-nCoV_N1-P**	200nM	5'-FAM-ACC CCG CAT/ZEN™/ TAC GTT TGG TGG ACC-3IABkFQ-3'	

F: forward; R: reverse

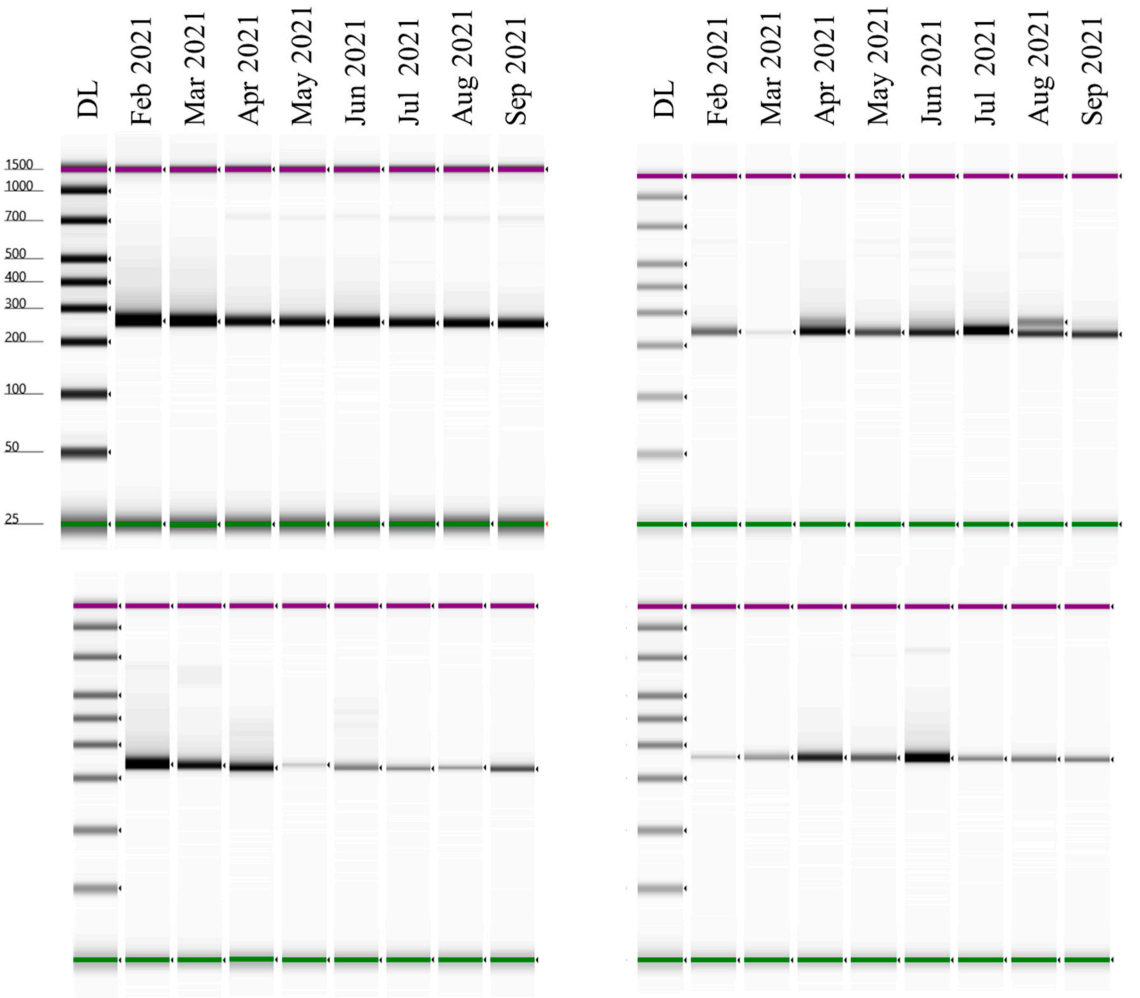

Figure S1: Amplicons from SARS-CoV-2 S gene from region 1 (top left), region 2 (top right), region 3 (bottom left) and region 4 (bottom right) were visualized in 4150 Tapestation System using the Agilent High Sensitivity D1000 ScreenTape Kit. Arrows indicate specific bands located between 200-300 bp, upper and lower markers, and digital ladder (DL).
